# Infection Rates by Dengue Virus in Mosquitoes and the Influence of Temperature May Be Related to Different Endemicity Patterns in Three Colombian Cities

**DOI:** 10.3390/ijerph13070734

**Published:** 2016-07-21

**Authors:** Víctor Hugo Peña-García, Omar Triana-Chávez, Ana María Mejía-Jaramillo, Francisco J. Díaz, Andrés Gómez-Palacio, Sair Arboleda-Sánchez

**Affiliations:** 1 Grupo de Biología y Control de Enfermedades Infecciosas, Universidad de Antioquia, Sede de Investigaciones Universitarias (SIU), Calle 62 # 52–59 Laboratory 620, P.O. Box: 1226, Medellín 050010, Colombia; victorhugopega@gmail.com (V.H.P.-G.); otriana@gmail.com (O.T.-C.); anamejia25@gmail.com (A.M.M.-J.); amgomezpa@gmail.com (A.G.-P.); 2 Grupo de Inmunovirología, Universidad de Antioquia, Sede de Investigaciones Universitarias, SIU, Calle 62 # 52–59 Laboratory 532, P.O. Box: 1226, Medellín 050010, Colombia; franciscodiaz314@gmail.com

**Keywords:** *Aedes aegypti*, dengue virus, infection rates, Breteau index, Colombia, climatic variables

## Abstract

Colombia is an endemic country for dengue fever where the four serotypes of virus dengue (DENV1–4) circulate simultaneously, and all types are responsible for dengue cases in the country. The control strategies are guided by entomological surveillance. However, heterogeneity in aedic indices is not well correlated with the incidence of the disease in cities such as Riohacha, Bello and Villavicencio. As an alternative, molecular detection of dengue virus in mosquitoes has been proposed as a useful tool for epidemiological surveillance and identification of serotypes circulating in field. We conducted a spatiotemporal fieldwork in these cities to capture adult mosquitoes to assess vector infection and explain the differences between Breteau indices and disease incidence. DENV infection in females and DENV serotype identification were evaluated and infection rates (IR) were estimated. The relationship between density, dengue cases and vector index was also estimated with logistic regression modeling and Pearson’s correlation coefficient. The lack of association between aedic indices and dengue incidence is in agreement with the weak associations between the density of the mosquitoes and their infection with DENV in the three cities. However, association was evident between the IR and dengue cases in Villavicencio. Furthermore, we found important negative associations between temperature and lag time from two to six weeks in Riohacha. We conclude that density of mosquitoes is not a good predictor of dengue cases. Instead, IR and temperature might explain better such heterogeneity.

## 1. Introduction

Dengue is a mosquito-borne infection that is present in tropical and subtropical regions around the world comprising more than 100 countries. It is estimated that 390 million new infections occur per year [1]. There is no available vaccine for dengue, and prevention and control of the disease have been focused on the elimination of *Aedes aegypti* populations [2], which is the most important dengue vector species.

Therefore, as a strategy of dengue surveillance, the World Health Organization (WHO) recommends routine estimation, among other things, of aedic larval indices in areas with active transmission [2]. Those indices include the house index (i.e., the ratio between positive houses and total sampled houses), the container index (i.e., the ratio between positive containers and total containers), and the Breteau index (i.e., the ratio between positive containers and 100 sampled houses). The last index is considered the most informative because it establishes the relation between the positive containers and houses [2]. However, some authors have found incongruences between those indices and the number of reported dengue cases [3,4,5], indicating that these indices do not necessarily reflect the status of mosquito infection or adult abundance [6,7,8].

As an alternative, molecular detection of dengue virus in mosquitoes has been proposed as a useful tool for epidemiological surveillance and identification of serotypes circulating in field [9,10,11,12]. Some approaches have been developed by the use of infection rates (IR) which, in turn, like other variables involved in transmission, might be affected by climatic variables such as temperature [13]. Virological surveillance through molecular methods has been proposed as a more informative and sensitive tool for assessing vector infection [9,10,11,14,15] and, therefore, the infection risk to human populations. Sensitive primers used in detecting dengue virus (DENV) have been mainly developed using sera from infected patients and mosquito samples [16,17,18,19,20,21,22,23,24]. The main characteristic of molecular methods with regard to serology and virus isolation is that they are fast, easy to use, and allow the detection of the four serotypes of DENV [16], which is important in areas where more than one serotype circulates. Thus, the transmission dynamics of DENV and its serotypes can be monitored in human and mosquito populations to predict and control new dengue outbreaks.

In addition, the weather is a key factor to have in mind in the epidemiological surveillance of dengue, since it affects some mosquito life traits as well as virus replication [25]. Thus, some features such as temperature, precipitation and humidity have been associated to mosquito development, survival, density and oviposition rates [25,26,27,28,29,30]. On the other hand, virus replication and transmission have been described as temperature-dependent [31,32,33,34]. However, the effect of climatic variables is not always linear, as the temperature at which the highest possible density of mosquitoes [26,35] and virus transmission [13,34] occurs can range between 15 °C and 32 °C.

Colombia is an endemic country for dengue with several epidemiological landscapes, where the four serotypes circulate and are responsible for multiple cases of disease [36,37]. In 2013, Colombia experienced an increase in morbidity (63,059 confirmed cases against 26,693 in 2012), which was declared an epidemic year [37,38]. However, since 2012 the vast heterogeneity in the epidemiology of dengue in Colombia has been considered as part of the problem. One example is the analysis of epidemiological and entomological data from three cities for that year: Riohacha showed high values for the Breteau indices (23.43) but low incidence (38.42 per 100,000 population); Bello had very low values for the Breteau index (4) and an incidence of 28.84 per 100,000 population; and Villavicencio had the highest incidence (466.1 per 100,000 population) with a Breteau index of 16.15. These three cities are endemic for dengue, and an incremental increase in dengue cases has been observed in the last six years [39].

Previous data suggest that the epidemiological trend in these cities is not well correlated with their entomological behavior. For that reason, in this study, we propose virological surveillance in mosquitoes as an additional parameter to be taken into account as a monitoring and control measure for dengue. Moreover, we study the relationship between dengue infection in mosquitoes with their density, and the main weather variables such as temperature, relative humidity and precipitation as well as its influence in human cases.

## 2. Materials and Methods

### 2.1. Study Sites and Field Work

**Figure 1:**
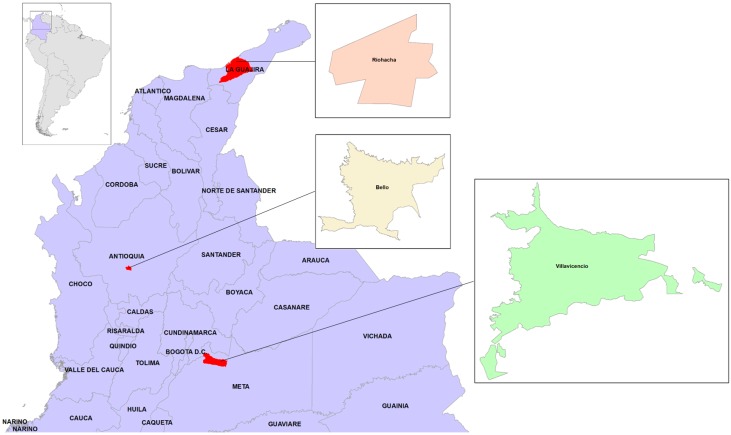
Geographic location of the three study cities in Colombia. The urbanized area of the cities is magnified.

This study was conducted in three Colombian cities: Riohacha, Bello and Villavicencio (Figure 1).

Riohacha is located in the La Guajira department (See File S1.kmz for detailed sampled locations) in Northern Colombia. The city is bounded by the Caribbean Sea to the Northwest. The average temperature is 27.9 °C with averaged minimum and maximum temperatures of 24.6 °C and 33.2 °C, respectively. The annual average relative humidity is 78.3%, and the annual precipitation is 920.3 mm with scarce rainfall from September to December. The estimated human population in 2012 was 231,641 inhabitants according to the Departamento Administrativo Nacional de Estadística [40]. In 2012, the average value for the Breteau index was 23.43 positive containers per 100 houses; however, some neighborhoods reached values of more than 100.

Bello is located in the Aburrá Valley, in the Department of Antioquia at 1450 meters above sea level (File S1). The average temperature is 22.2 °C, and the averaged minimum and maximum temperatures are 17.3 °C and 29 °C, respectively. The annual precipitation is 1850 mm, and the average relative humidity is 73%. The population in 2012 was estimated to be 429,984 inhabitants [40]. In 2012, the average value of the Breteau index was 4 positive containers per 100 houses.

Villavicencio is located in the Meta Department at the foothills of the Andean mountains where the eastern plains of Colombia begin at 467 meters above sea level (File S1). The average temperature is 26 °C with averaged minimum and maximum temperatures of 21.2 °C and 30.8 °C, respectively. The mean annual precipitation is 4545 mm, and the relative humidity is 74%. The total population in 2012 was 452,472 [40]. The average value of the Breteau index in 2012 was 16.15 positive containers per 100 houses, reaching values of 86.96 in some places.

We sampled four neighborhoods from each city from June 2012 to December 2013, and every neighborhood was sampled between 3 and 5 times. The fieldwork in a city to capture DENV transmitting-mosquitoes at a given time is defined as a sampling. The sampling times were chosen after 15–30 days into the rainy season but also took into account the periodicity in mosquito populations throughout the year. At every sampling, we chose 20 houses randomly distributed throughout each neighburhood; therefore, each house (space) in a given sampling (time) was defined as an observational unit. Every house was visited twice per day at the same hours, morning and afternoon, during 3 to 5 consecutive days, and all adult mosquitoes seen inside the house were actively captured with entomological nets by searching in bedrooms, bathrooms, living rooms, and other places inside the houses where inhabitants reported its presence. The searching activity inside each house lasted at least 15 min. The staff that conducted the capture activities was trained in searching mosquitoes, sexing, handling and identification of mosquitoes at genus level in field. The same personnel performed capture activities in the three cities and the same neighborhoods across the entire study time.

Mosquitoes were sexed, and females were pooled according to the observational unit and conserved in RNAlater^®^ (AMBION, Inc., Austin, TX, USA). The taxonomic classification of the mosquitoes was confirmed by using the Walter Reed Army Institute for Research taxonomical key developed by Rueda (2004) [41]. Finally, samples were stored at −70 °C until needed. Every house was georeferenced using a Global Positioning System (GPS, Garmin GPSMAP^®^ 62s, Olathe, KS, USA) with 30 m of precision.

Information about the Breteau indices was provided by local health authorities from each city. These health authorities conduct entomological surveillance across each city three to four times per year, following the guidelines of the World Health Organization [2].

### 2.2. Experimental Infection of Mosquitoes

Mosquitoes from the Rockefeller colony were experimentally infected with each of the four serotypes of DENV to be used in Polymerase Chain Reaction (PCR) standardization and to obtain positive controls for diagnostic PCRs. The strains used included the Western Pacific belonging to DENV-1, New Guinea C (NGC) for DENV-2, H-87 for DENV-3 and H-241 for DENV-4. Because infected mosquitoes were used as positive controls, we experimentally infected mosquitoes through microinjection to ensure 100% infection. Briefly, mosquitoes were anesthetized in cold temperatures and intrathoracically injected with virus using a borosilicate glass capillary needle attached to a syringe with a 3-way stopcock through plastic tubing, according to methodology reported by Medina and co-workers [42].

### 2.3. RNA Extraction

Because the power of detection of our used methodology was standardized with a maximum of 20 mosquitoes (see next section), RNA extraction was conducted in pools ranging from 1 to 20 mosquitoes collected from field, and also in individual mosquitoes that were experimentally infected. When a greater number of mosquitoes were captured inside one house, the pool was divided in halves and analyzed separately.

Total RNA was extracted from mosquitoes using Qiazol^®^ lysis reagent (Qiagen, Hilden, Germany), following the manufacturer’s instructions, with prior homogenization of mosquitoes using a pestle in 1.5 mL tubes, followed by centrifugation at 12,000 rpm for 10 min. Once RNA was obtained, its concentration and quality were measured using a Nanodrop-2000 spectrophotometer (Thermo Fisher Scientific, Waltham, MA, USA).

### 2.4. Molecular Detection of Virus in Mosquitoes

To detect DENV in the field mosquitoes, we first carried out a series of experiments to choose the set of primers with the best detection limit for our samples. We selected four sets of primers designed by Chutinimitkul et al. (pair A) [43], Shu et al. (pair B) [18], Chien et al. (pair C) [20], and Seah et al. (pair D) [17,44], which are listed in Table S1. Every set of primers was tested with two protocols. The first protocol consisted of using the QuantiTect SYBR^®^ green RT-PCR kit (Qiagen) according to the manufacturer’s instructions. The second one was an in-house protocol as follows: For the pair A primers, we used MgCl2 at 1.5 mM, dNTPs 0.2 mM, primers 0.5 µM, SYBR^®^ Green 0.5X, TrueStart Hot Start polymerase (Thermo Fisher Scientific) 0.5 U, Buffer TrueStart (Thermo Fisher Scientific) 1X, MMuLV 25 U and 3 µL of RNA. For the pair B primers, we used the same conditions described for pair A but with 3 mM of MgCl_2_, 0.6 µM of primers and 0.25X of SYBR^®^ Green. For the pair C primers, we used the same conditions as used for pair A but only 0.15 µM of primers. Because pair C primers were designed for a hemi-nested PCR, we added 2 µL of amplicon to the second PCR with the same conditions. For pair D primers, we used the same conditions as used for pair B but added 2 mM of MgCl_2_ and 0.25X of SYBR^®^ Green. The thermal profile for each set of primers was identical to the one reported by the authors.

To test the detection limit of each primer set, RNA from experimentally infected mosquitoes was serially diluted from 10 ng/µL to 0.01 ng/µL. Additionally, we used pools with 5, 10, and 20 mosquitoes containing a single infected mosquito, and the RNA extracted from every pool was diluted to 100, 50 and 10 ng/µL. In both cases, the PCR was performed as described above.

### 2.5. Detection of Dengue Virus and Serotype Identification in Field-Captured Mosquitoes

Field-captured *Ae. aegypti* females were analyzed in pools consisting of no more than 20 mosquitoes, according to the previous description. PCRs were performed as multiplex RT-PCRs using the commercial kit QuantiTect SYBR^®^ Green RT-PCR kit (QIAGEN) but modifying the volume to 15 µL. When one sample was positive by RT-PCR, the serotype was confirmed by running the sample in a second RT-PCR using the forward primer and one single serotype-specific reverse primer. The PCR sample was loaded into a 1.5% agarose gel stained with ethidium bromide to confirm the band size.

### 2.6. Epidemiological Data

Data on dengue cases from each city were obtained from the Sistema Nacional de Vigilancia en Salud Pública (SIVIGILA), a Colombian system to collect and systematically provide timely information on events related to public health. The weekly information is available through the Instituto Nacional de Salud (INS, Bogotá, Colombia; [45]).

### 2.7. Climate Data

Data on daily temperature, precipitation and relative humidity were obtained from the Colombian Institute of Hydrology, Meteorology and Environmental Studies (IDEAM). Data used in this study were recorded in meteorological stations located at the Airport Almirante Padilla for Riohacha city, Vanguardia airport for Villavicencio city, and at Tulio Ospina station for Bello city. Daily averages of the three evaluated variables were calculated weekly for use in analysis.

### 2.8. Statistical Analyses

To test if epidemiological differences between cities were correlated with infection in mosquitoes rather than mosquito density, we first tested for significant differences in mosquito density. For this, a generalized linear model (GLM) was built with data corresponding to the number of mosquitoes captured in each city, using number of mosquitoes as the dependent variable and city as the independent variable. We assumed that the same sampling effort was conducted in the three cities as mentioned above. Analyses were performed based on a Poisson distribution for counting data with a logarithmic link function. R software v.3.1.0 (R Foundation for Statistical Computing; Vienna, Austria) was used in the development of this analysis.

To estimate the relationship between adult mosquito density and mosquito risk of infection based on infected vectors captured inside houses, a logistic regression model (LRM) was conducted using a binomial distribution with logit as the link function with infection of mosquitoes as the dependent variable and number of mosquitoes per house as the independent variable. The analysis was conducted using the data as observational units without taking into account the number of pools analyzed in molecular tests, since inside some houses we trapped more than 20 mosquitoes and subsequently we divided them to be tested into two pools. This analysis was performed using Base package of R Software.

IR were estimated with the maximum likelihood estimator (MLE) method for unequal pool sizes [46] through the function pooledBin implemented in the binGroup R package. The parameter used to estimate the IR was a bias-corrected MLE with a skewness-corrected confidence interval. All calculations were performed at the scale of 1000 mosquitoes.

In addition, a vector index (VI) was calculated to estimate the average number of DENV-infected mosquitoes per sampled observational unit for single species (*Ae. aegypti*). The VI is calculated by multiplying the abundance of captured mosquitoes per observational unit with the estimated proportion of infected females. The VI was originally used in studies of surveillance of West Nile virus as a measure of abundance of infected mosquitoes using trap collection data [47,48]. We used data from the active capture developed by our personnel to estimate VI. Calculations were done according to Center for Disease Control and Prevention (CDC) guidelines [49].

We used Pearson’s correlation coefficient analyses to find correlations between the mosquito IR with dengue cases and climatic variables. Chan and Johanson (2012) [33] estimated an extrinsic incubation period at 25 °C from 5 days (95% confidence interval (CI): 3–8 days) to 33 days (95% CI: 23–48 days) and an intrinsic incubation period from 3.4 days (95% CI: 3.0–3.7 days) to 10 days (95% CI: 9–11 days). Therefore, we took into account: (a) the weekly averages of epidemiological data during the same sampling week (listed as 0) and up to six weeks before (listed from −1 to −6) sampling for climatic variables; and (b) the epidemiological period, which is defined as four consecutive epidemiological weeks, and the epidemiological weeks before sampling (listed from −1 to −3) and after (listed from 1 to 6) for dengue cases.

Data management was conducted through Microsoft Excel 10 (Microsoft Corporation; Redmond, Washington, DC, USA), and the graphs were built using GraphPad Prism 5 (GraphPad Software, Inc., La Jolla, CA, USA).

## 3. Results

### 3.1. Field-Captured Mosquitoes Support the Breteau Indices

In total, 2107 *Ae. Aegypti* were captured; 897 (42.57%) males and 1210 (57.43%) females. Of these, 42.07% were collected in Riohacha, 39.42% in Villavicencio, and 18.51% in Bello (Table 1). The average numbers of mosquitoes captured inside houses were 3.77, 3.33, and 2.36 mosquitoes per positive house from Riohacha, Villavicencio and Bello, respectively. Differences in the average number of mosquitoes inside houses (*p* < 0.05) between the three cities are coherent with global Breteau indices reported and detailed previously.

### 3.2. Molecular Detection of DENV in Experimentally Infected Mosquitoes

The PCRs were carried out with the kit reagents that showed the best amplification quality in comparison to in-house PCRs. The best PCR detection limit was reached using the set B primers designed by Shu and co-workers (2003) [18], which detected the four serotypes of the virus at 0.01 ng/µL. (Figure S1). The PCR using the other primers failed to detect some serotype(s) at the lowest RNA concentrations (Figure S1). Set B primers and kit reagents could detect one infected mosquito in a pool of 20 in the three dilutions. These conditions were used posterior to PCR reactions.

### 3.3. Dengue Infection in Mosquitoes Is Not Related to Mosquito Density

The females were grouped into 373 pools, each comprising from 1 to 19 mosquitoes. From these, 45 (12.06%) of these pools were positive for DENV infection. The highest percentage of positive pools was found in Bello (16.84%), followed by Villavicencio (11.19%) and Riohacha (9.63%). The results per neighborhood showed similar values (Table 1). The LRM model indicated a low (3.98%) correlation between houses with infected mosquitoes (response variable) and the density of mosquitoes per house (explanatory variable) (*p* = 0.001). Similar results were obtained by modeling every city separately (Table S2).

### 3.4. Infection Rate (IR) of Field-Captured Mosquitoes, Vector Index (VI) and Its Relation to Dengue Cases

**Figure 2:**
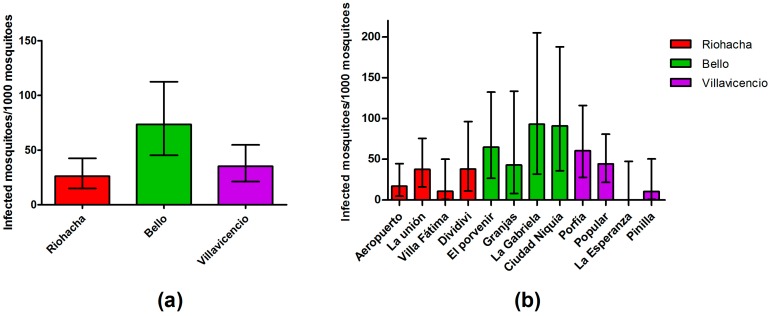
IR estimated with upper and lower limit (error bars) from mosquitoes for: each city (**a**); and each neighborhood (**b**).

The estimated IR for all datasets was 38.55 infected mosquitoes per 1000 mosquitoes. Bello showed the highest infection rate, followed by Villavicencio and Riohacha (Figure 2a). When estimations were done at the neighborhood level, the same pattern was found, except for the neighborhood La Esperanza (Villavicencio), which did not show any infected mosquitoes (Figure 2b).

**Figure 3:**
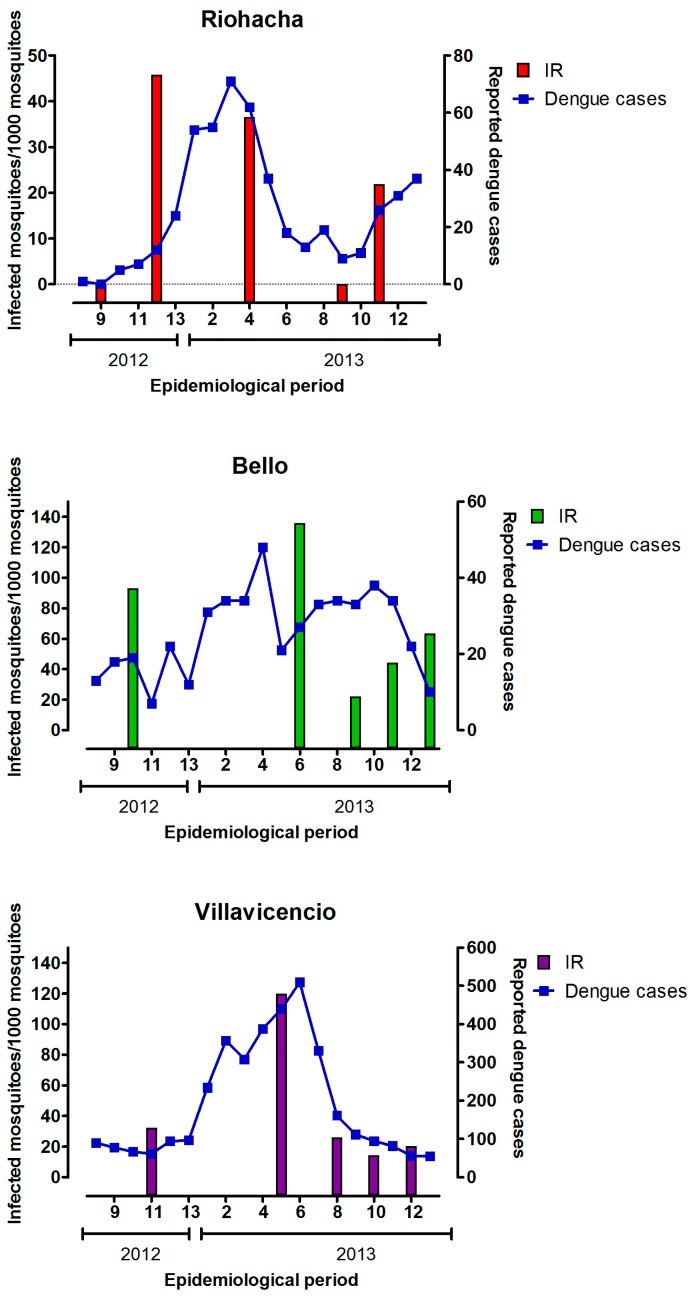
Behavior of dengue cases notified by INS (blue line) and IR of mosquitoes (bars) through the time of study (mid-2012 and 2013). Epidemiological period and year are displayed in the x-axis.

To correlate the IR with dengue cases in each city, we considered the epidemiological period and the same and previous epidemiological weeks in which every sampling was conducted. A similar pattern was observed in the three cities where an increase in dengue cases was observed through year 2013, and this pattern disappears abruptly at the epidemiological period number nine (Figure 3). However, the results of the Pearson analysis showed that a positive correlation between IR and dengue cases exists in Villavicencio, but not in Riohacha and Bello (Table S3).

The information related to dengue cases is provided by INS to epidemiological week level. In this way, we explored the possibility that the IR could be correlated with dengue cases some weeks before (indicating transmission from human to mosquitoes) or after (indicating transmission from mosquitoes to humans) the weeks of samplings. We found a positive correlation between IR and dengue cases at one week before, the same week and one to six weeks after samplings in Villavicencio (Table S3).

Finally, the same analyses were done with VI. We tested whether VI better predicts the dengue cases than infection rates by examining whether VI improves correlations. If so, density might play a role in the outcome of dengue cases. However, the analysis showed less significant correlations with Villavicencio and some correlations with infection rates in the same city were lost when the correlations were made with vector index (Table S4).

### 3.5. Dengue Serotypes Circulating in Field Mosquitoes

**Figure 4:**
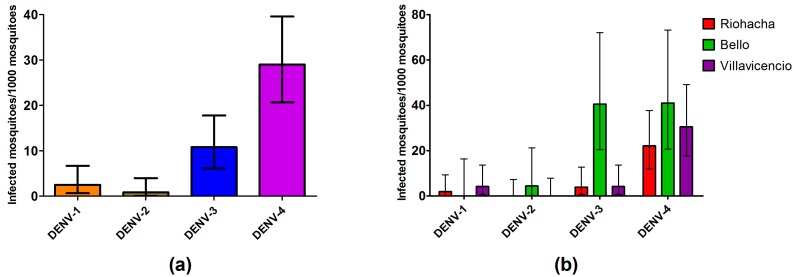
IR estimated with upper and lower limit (error bars) from mosquitoes for: each serotype (**a**); and serotypes discriminating by city (**b**).

Serotype DENV-4 was detected in 75.5% of the samples, resulting in the highest infection rate per serotype (29 mosquitoes per 1000 mosquitoes) (Figure 4a), while DENV-3 was detected in 28.8%, DENV-2 in 2.22% and DENV-1 in only 6.66% (Table S5). Six samples showed mixed infection with two serotypes (13.3%), which were detected in 7.69% of the Riohacha positive pools, 18.75% of the Bello, and 12.5% of the Villavicencio (Table S5). Two of the pools containing only one mosquito showed mixed infection, one of them with serotypes DENV-4 and DENV-1 (from Villavicencio) and the other one with DENV-4 and DENV-3 (from Bello). Serotypes detected in Bello were DENV-2, DENV-3 and DENV-4, while in Villavicencio and Riohacha DENV-1, DENV-3 and DENV-4 were detected (Figure 4b).

### 3.6. Mosquitoes Infection Rate by Serotypes and Human Cases

Temporal analyses conducted to explore the correlation between the IR calculated in each sampling and the dengue case rates showed that DENV-3 was associated in Riohacha with case occurrence at Weeks −1, 0, and 1; in Villavicencio, DENV-3 was associated with cases that occurred at Weeks −3, −2, −1, 0, and 1; and in Bello, DENV-3 was not associated with the occurrence of dengue cases (Table S6). The DENV-2 serotype was associated with dengue only in Bello at Week −1. DENV-4 was associated with dengue in Riohacha at Week 6; in Villavicencio at Weeks −1, 0 and 1 to 6; and in Bello, there was no significant correlation (Table S6).

### 3.7. Mosquitoes Infection Rate and Climatic Variables

Significant isolated correlations were found in Bello between IR and precipitation at Week −4 and with temperature at Week −5. In Villavicencio, the IR was correlated with relative humidity at Week −3 and with temperature at Week −4 (Table S7). However, the most important correlations were found in Riohacha with relative humidity at Weeks −4, −5 and −6 and with temperature at Weeks −2, −4, −5 and −6 (the *p*-value at Week −3 was 0.058) (Table S7; Figure S2). According to these results, the relative humidity positively affects IR with a lag time of a month or more, and temperature negatively affects IR with a lag time of two to six weeks.

## 4. Discussion

Being a country endemic for dengue fever, Colombia expends a large amount of its resources on vector control activities. In that way, when an epidemic is ongoing, the local health authorities prioritize areas with the highest vector infestation to control the disease because high densities have been suggested to increase the DENV transmission risk. We did not find a direct relationship between mosquito density and mosquito infection by DENV; it is important to have in mind that we only sampled the adult populations, which should reflect the epidemiological situation in a particular area [7,8,50,51]. In this work, we showed that the infection status by DENV of the three mosquito populations from Colombia is not correlated with mosquito density, which is an idea put forward previously but has never been demonstrated [5,7,51]. In this respect, our results suggest that the infection dynamics in mosquitoes and, to some extent, the risk of infection in humans, needs to be clarified.

Differences we found in mosquito density between cities may be explained by local customs and service coverage. Riohacha has an insufficient coverage of water services, and therefore, residents must store water in large containers. The availability of contained water, coupled with high temperatures, can accelerate the development of mosquitoes [52,53,54,55], resulting in the high amount of vectors reported in that city. On the other hand, people from Villavicencio have similar customs; although the water coverage is 36% better there than in Riohacha (90.1% for Villavicencio against 54% for Riohacha), inhabitants store water for use in housework. Villavicencio has high precipitation and temperature, which also could explain the vector density in those places. In Bello, the water coverage and the garbage collection is adequate (95.1%), but in contrast to the residents in Riohacha and Villavicencio, people from Bello do not store water for housework; therefore, vases are the main mosquito breeding containers, which are a low charge container. Therefore, the breeding in this container in association with a medium temperature, could explain the low aedic indices in the city.

According to assumptions of control strategies guided by entomological indices, a higher amount of mosquitoes increases risk of dengue fever; hence, higher dengue cases should be expected in places with higher density of mosquitoes; however, we did not find a constant relation across time. During the time of the study (June 2012 to December 2013), Riohacha showed a low incidence (205.83 per 100,000 population) related to its higher Breteau index (23.43), and the lowest mosquitoes IR (26.08 per 1000 mosquitoes), while Bello showed a high number of dengue cases (112.65 per 100,000 population) related to its low Breteau index (4) and higher mosquitoes IR (73.53 per 1000 mosquitoes). Notably, the amount of mosquitoes captured in the cities seems to be congruent with the Breteau indices at the city level. This leads us to suggest that the lack of correlation between aedic indices and incidence is not explained by a lack of correlation between the abundance of the adults and the aedic indices. Rather, it is explained by other biological variables related to infection in mosquitoes. In that sense, the IR could offer insights on the differences in dengue cases reported among cities and the lack of relation between IR and the Breteau indices. It is important to note that larval indices are estimated indoor, which is exactly the same environment we sampled, it is probably that this is the reason why we find good concordance between Breteau index and the average number of females collected by house. This is important because it is a good indicator of an adequate sampling and that our conclusions are not related to different sampling environment.

It was very important to obtain a high performance in our detection system in order to reach a proper estimation of IR. Using primers reported by Shu [18], we were able to accurately estimate the highest IR reported in Colombia. Although Méndez and co-workers [56] estimated IR during an epidemic period in Colombia during 2002 and 2003, they reported an IR of 11.6. IR has a large range of values in the scientific literature, but few reports exceed the IR we recorded in Colombia [9,57], which may reflect the high performance of the primers.

Although molecular detection on field-captured mosquitoes does not allow us differentiate infected from infective mosquitoes, nor even the age or number of gonotrophic cycles, is remarkable that mosquito IR could be a dengue predictor in places such as Villavicencio where high number of dengue cases is constantly reported. Our results showed a high correlation between IR and dengue with a lag time ranging from three to six weeks. This lag time could suggest that cyclic dengue virus transmission is taking place every three weeks in this city, although this correlation depends on many factors such as, human immunity, because many cases are unrecognized [1,58]; pathogenicity of the virus strain [59]; and vectorial capacity [13]. Therefore, it is necessary to develop further studies to confirm cyclic behavior.

On another hand, VI was a weaker predictor than IR of dengue cases. VI is a concept that try to link the IR with density to provide a more complete panorama of transmission risk. To get this, IR is multiplied by density of mosquitoes and the result is the VI. However, the VI had fewer and less significant correlations with dengue cases than IR when it is used separately. The loss of significance in correlations of IR with dengue cases when IR is multiplied by density, confirms that the last is not playing a crucial role in transmission in the three cities.

Regarding the IR of individual serotypes vs. dengue cases, DENV-4 in mosquitoes shows a similar pattern to that displayed when serotype is not considered, which can be expected because DENV-4 was the most abundant in mosquitoes. However, DENV-3 in mosquitoes showed correlation with dengue cases in Riohacha and Villavicencio before samplings, suggesting an introduction event of some virulent genotype of DENV-3 into the susceptible population, which could be evidenced by the disease manifestation.

Our findings indicate that DENV-2, DENV-3, and DENV-4 circulated in mosquitos from Bello between 2012 and 2013, whereas DENV-1, DENV-3, and DENV-4 circulated in mosquitoes from Riohacha and Villavicencio in that same period. Accordingly, the possibility that a differential transmission of serotypes explains the different endemicity patterns should not be considered since Riohacha and Villavicencio share the same serotypes but have opposing endemic and entomological patterns.

In Riohacha, high temperatures might constrain infection development in mosquitoes, which could explain why Riohacha has few dengue cases but high vector densities. This correlation was present for only one week in Bello (Table S7), whereas in Riohacha it was persistent over five weeks. It seems that in Riohacha, the negative effect of temperature is strong and continuous over time. This has been observed in other studies, both experimentally and in the field. Liu-Helmersson [13] and Yang [26] suggest that temperatures above 32 °C can negatively affect DENV transmission, and Yu and co-workers [60] observed negative associations between temperatures over 30 °C and dengue cases in tropical regions of Taiwan. In 2012 and 2013, the highest recorded temperatures in Riohacha were 38 °C and 39.4 °C, respectively, with a monthly maximum average of 33.5 °C for 2012 and 33.8 °C for 2013, thus far exceeding 30 °C. Furthermore, negative effects of temperature on viral infection in mosquitoes have been experimentally observed with other arboviruses [61,62,63,64]. The mechanisms behind this phenomenon are not well characterized, but a few pieces of evidence exist. Zhang [65], for example, described differential conformation of virus particles when they are at 28 °C versus at 37 °C.; at 37 °C, the virus particle adopts a “bumpy” conformation, which probably facilitates the course of infection in humans; on the other hand, a smooth particle conformation adopted at 28 °C could be optimal for mosquito infection and dissemination [65,66]. High environmental temperatures probably cause virus particles to adopt the “wrong” conformation, thus affecting mosquito infection. Additionally, Adelman [67] demonstrated that cooler environmental temperatures affect the functioning of iRNA machinery, an important immune pathway in mosquitoes, which increases the infection levels. Although they only tested up to 28 °C, it is likely that higher performance of proteins implicated in this immune route is achieved at higher temperatures. Alternatively, Goindin and co-workers (2015) [68] estimated a decrease in mosquito life expectancy and subsequent decrease in the infective life expectancy as a result of an increase in temperatures.

Some associations were observed in Riohacha and Bello between precipitation and IR, but not in Villavicencio. Since the relationship of rainfall to dengue is mainly associated with a greater availability of mosquito breeding places, but the local habit in Villavicencio of storing water results in the availability of breeding places with no need of rain. This fact highlights the importance of good water coverage services and sewage system in the cities, since it directly impacts public health. Also, it is important to note how local customs can impact the outcome of the disease, which jointly with weather and service coverage, lead to the disease being a result of multiple factors unique to each city.

Finally, although the influence of weather on dengue cases and mosquitoes at the laboratory level has been demonstrated extensively (reviewed by Morin [25]), this, to our knowledge, is the first report of a direct effect of temperature on the IR of naturally infected mosquitoes from field. This is an important step in the understanding of transmission on a fine scale, i.e., without assuming what happens between temperature and the final number of dengue cases. We encourage further studies to fill the gaps in the transmission and outbreaks events.

## 5. Conclusions

DENV transmission dynamics cannot be explained by mosquito density alone. A complex network of variables is involved in the process, including vectorial capacity, heterogeneity in human immunity, and abiotic variables such as temperature. An inconsistent relationship between mosquito IR and dengue cases reflects the complexity in vectorial capacity and human immune mechanisms, and this emphasizes the importance of including viruses circulating in both humans and mosquitoes in epidemiological surveillance. Weather variables can influence the development and infection of the virus and its posterior transmission, and this varies across locations. Thus, it is necessary to incorporate all of these fine scale dynamics when developing and implementing dengue control strategies.

## Figures and Tables

**Table 1:** Number of captured mosquitoes by neighborhood and pools analyzed for percent positivity.

City	Neighborhood	Females Captured	Number of Pools	Positive Pools (%)
Riohacha	Aeropuerto	175	44	3 (6.82)
	La Unión	167	46	6 (13.04)
	Villa Fátima	90	23	1 (4.35)
	Dividivi	77	22	3 (13.64)
Bello	El Porvenir	77	24	5 (20.83)
	Granjas	47	20	2 (10)
	La Gabriela	43	27	4 (14.81)
	Ciudad Niquía	57	24	5 (20.83)
Villavicencio	Porfía	128	40	7 (17.5)
	Popular	185	58	8 (13.79)
	La Esperanza	70	21	0 (0)
	Pinilla	94	24	1 (4.17)
Total		1210	373	45 (12.06)

## Supplementary Materials

The following are available online at www.mdpi.com/1660-4601/13/7/734/s1, Figure S1. Detection limit of three different sets of primers for detecting virus in mosquitoes at different concentrations of total RNA. The concentration of the total RNA is indicated above each lane. The corresponding serotype of the virus is indicated to the right of each gel. The set of primers used is indicated on the left. Figure S2. Relationship between IR and temperature at different weeks before samplings in Riohacha. (a) Relationship between IR and the temperature six weeks before; (b) five weeks before; (c) four weeks before; (d) three weeks before; (e) two weeks before and (f) One weeks before. (a–c) and (e) were significant with 95% confidence. D was significant with 94% of confidence. Table S1. Primers used in this study. Table S2. Results of modelling of the positivity of mosquitoes for dengue virus infection and the density of mosquitoes per house for the three cities (Global) and each city separately. Table S3. Correlation between IR and dengue cases at different weeks after and before samplings. Correlation coefficients for IR and dengue cases are shown for the epidemiological period and every week from three weeks before (−3, −2, and −1) until six weeks after (1, 2, 3, 4, 5, and 6) every sampling. Significant correlation coefficients are shown in bold. Table S4. Correlation between VI and dengue cases at different weeks after and before samplings. Correlation coefficients for VI and dengue cases are shown for the epidemiological period and every week from three weeks before (−3, −2, and −1) until six weeks after (1 to 6) every sampling. Significant correlation coefficients are shown in bold. Table S5. Number of positive pools for each serotype for the three cities. Pools with mixed infection are indicated as DENV-1/4 (with serotypes DENV-1 and DENV-4) and DENV-3/4 (with serotypes DENV-3 and DENV-4). Table S6. Correlation between IR for each dengue serotype and dengue cases. Correlation coefficients for IR of every serotype are shown for epidemiological period and every week from three weeks before (−3, −2, and −1) until six weeks after (1, 2, 3, 4, 5, and 6) every sampling. Significant correlation coefficients are shown in bold. Table S7. Correlation between IR and climatic variables. Correlation coefficient for IR and different climatic variables are shown for every week from six weeks before (−6 to −1) to the same week of the samplings. Significant correlation coefficients are shown in bold. File S1: Location of sampled neighborhoods of Riohacha (northern city), Bello and Villavicencio (southern city). The KMZ file is composed of individual .kml files of each neighborhood consisting of place markers for the center of the neighborhood. Every .kml file is named according to the name of the neighborhood.

## Abbreviations

The following abbreviations are used in this manuscript:

DENV	Dengue virus
WHO	World Health Organization
GLM	Generalized linear model
LRM	Logistic regression model
IR	Infection rates
VI	Vector index
