# Supplementary material for: Infection Rates by Dengue Virus in Mosquitoes and the Influence of Temperature May Be Related to Different Endemicity Patterns in Three Colombian Cities
Source: Int J Environ Res Public Health. 2016 Jul 21;13(7):734. doi: 10.3390/ijerph13070734 (PMC4962275; doi:10.3390/ijerph13070734)
Supplement: Supplementary file 1 [file ijerph-13-00734-s001.pdf]

Supplementary Materials: Infection Rates by Dengue Virus in Mosquitoes and the Influence of Temperature May Be Related to Different Endemicity Patterns in Three Colombian Cities

Víctor Hugo Peña-García, Omar Triana-Chávez Ana María Mejía-Jaramillo, Francisco J. Díaz, Andrés Gómez-Palacio and Sair Arboleda-Sánchez

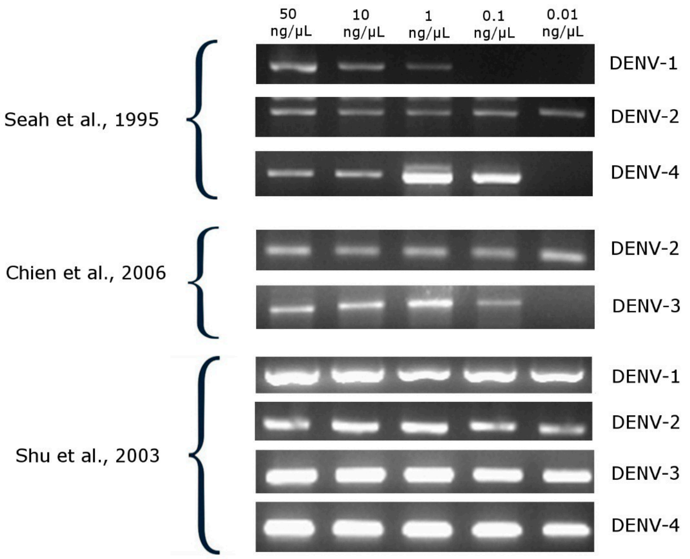

Figure S1. Detection limit of three different sets of primers for detecting virus in mosquitoes at different concentrations of total RNA. The concentration of the total RNA is indicated above each lane. The corresponding serotype of the virus is indicated to the right of each gel. The set of primers used is indicated on the left.

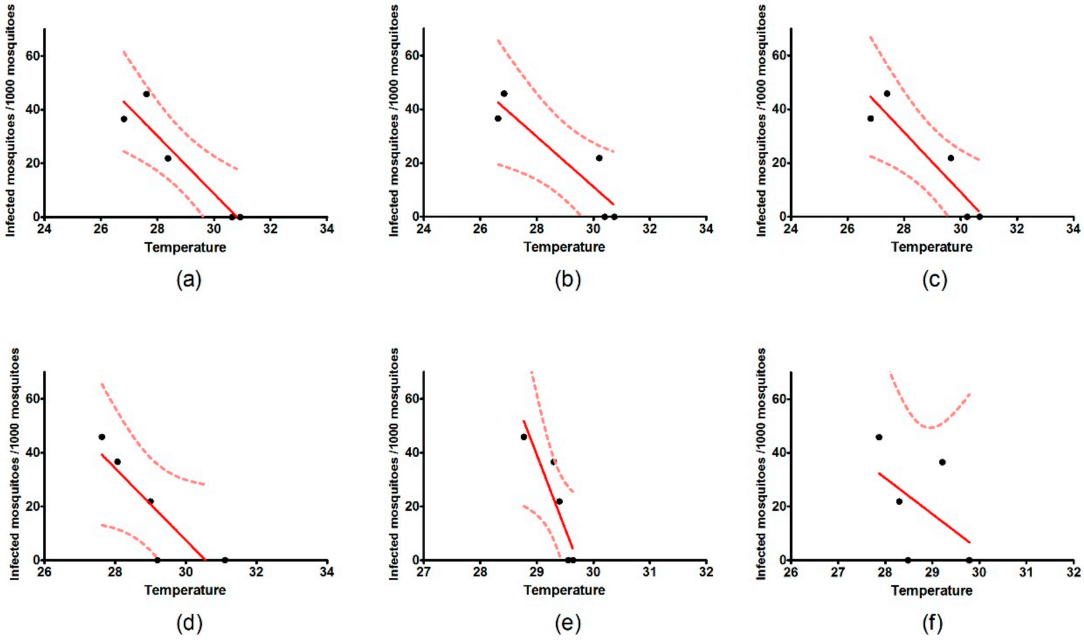

Figure S2. Relationship between IR and temperature at different weeks before samplings in Riohacha. (**a**) Relationship between IR and the temperature six weeks before; (**b**) five weeks before; (**c**) four weeks before; (**d**) three weeks before; (**e**) two weeks before and (**f**) One weeks before. (**a**–**c**) and (**e**) were significant with 95% confidence. D was significant with 94% of confidence.

Table S1. Primers used in this study.

Author	Sequence	Genome Region	Letter
Chutinimitkul et al., 2005 [1]	DEN_F: 5′-TTAGAGGAGACCCCTCCC-3′	3′UTR	A
	DEN_R: 5′-TCTCCTCTAACCTCTAGTCC-3′		
Shu et al., 2003 [2]	DN-F: 5′-CAATATGCTGAAACGCGAGAGAAA-3′	C gene	B
	DN-R: 5′-CCCCATCTATTCAGAATCCCTGCT-3′		
	D1-R: 5′-CGCTCCATACATCTTGAATGAG-3′		
	D2-R: 5′-AAGACATTGATGGCTTTTGA-3′		
	D3-R: 5′-AAGACGTAAATAGCCCCCGAC-3′		
	D4-R: 5′-AGGACTCGCAAAAACGTGATGAAT-3′		
Chien et al., 2006 [3]	mD1: 5′-TCAATATGCTGAAACGCGAGAGAAACCG-3′	C and prM genes	C
	D2: 5′-TTGCACCAACAGTCAATGTCTTCAGGTTC-3′		
	rTS1: 5′-CCCGTAACACTTTGATCGCT-3′		
	mTS2: 5′-CGCCACAAGGGCCATGAACAGTTT-3′		
	TS3: 5′-TAACATCATCATGAGACAGAGC-3′		
	rTS4: 5′-TTCTCCCGTTCAGGATGTTC-3′		
Seah et al., 1995a, 1995b [4,5]	DV1: 5′-GGRACKTCAGGWTCTCC-3′	NS3 gene	D
	DV3: 5′-AARTGIGCYTCRTCCAT-3′		
	DSP1: 5′-AGTTTCTTTTCCTAAACACCTCG-3′		
	DSP2: 5′-CCGGTGTGCTCRGCYCTGAT-3′		
	DSP3: 5′-TTAGAGTYCTTAAGCGTCTCTTG-3′		
	DSP4: 5′-CCTGGTTGATGACAAAAGTCTTG-3′		

Table S2. Results of modelling of the positivity of mosquitoes for dengue virus infection and the density of mosquitoes per house for the three cities (Global) and each city separately.

Locality	Model p Value	AIC	D^2
Global	0.001	355.55	3.98
Riohacha	0.1	111.48	2.05
Bello	0.01	127.05	4.99
Villavicencio	0.01	121.63	6.87

AIC: Akaike information criterion; D^2: Deviance statistics.

Table S3. Correlation between IR and dengue cases at different weeks after and before samplings. Correlation coefficients for IR and dengue cases are shown for the epidemiological period and every week from three weeks before (−3, −2, and −1) until six weeks after (1, 2, 3, 4, 5, and 6) every sampling. Significant correlation coefficients are shown in bold.

Week	City		
	Riohacha	Bello	Villavicencio
EP †	0.54769	−0.2893	**0.95367** *
−3	0.18616	−0.58975	0.66255
−2	0.74355	−0.4231	0.73745
−1	0.56743	−0.02439	**0.91216** *
0	0.40826	−0.17318	**0.95061** *
1	0.41422	−0.16282	**0.94938** *
2	0.59887	−0.12184	**0.97914** **
3	0.72141	0.22024	**0.99226** ***
4	0.75852	−0.46373	**0.98667** **
5	0.71735	-0.44176	**0.98571** **
6	0.80012	-0.19248	**0.99178** ***

* p-value < 0.05; ** p-value < 0.01; *** p-value < 0.001; † EP: Epidemiological Period.

Table S4. Correlation between VI and dengue cases at different weeks after and before samplings. Correlation coefficients for VI and dengue cases are shown for the epidemiological period and every week from three weeks before (−3, −2, and −1) until six weeks after (1 to 6) every sampling. Significant correlation coefficients are shown in bold.

Week	City		
	Riohacha	Bello	Villavicencio
EP †	0.27401	−0.21332	0.87053
−3	−0.05116	−0.63909	0.52646
−2	0.51944	−0.01770	0.60842
−1	0.29710	−0.10244	0.81084
0	0.123	0.08283	0.87118
1	0.14396	−0.01371	0.86761
2	0.39533	−0.09986	**0.91650** *
3	0.50394	0.33780	**0.94644** *
4	0.73532	−0.14183	**0.9296** *
5	0.6671	−0.11229	**0.95704** *
6	**0.93091** *	0.11021	**0.93892** *

* *p*-value < 0.05; † EP: Epidemiological Period.

Table S5. Number of positive pools for each serotype for the three cities. Pools with mixed infection are indicated as DENV-1/4 (with serotypes DENV-1 and DENV-4) and DENV-3/4 (with serotypes DENV-3 and DENV-4).

City	DENV-1	DENV-2	DENV-3	DENV-4	DENV-1/4	DENV-3/4	Total
Riohacha	1	0	1	10	0	1	13
Bello	0	1	6	6	0	3 *	16
Villavicencio	1	0	1	12	1 *	1	16
Total	2	1	8	28	1	5	45

* Pool composed by one mosquito infected with two serotypes.

Table S6. Correlation between IR for each dengue serotype and dengue cases. Correlation coefficients for IR of every serotype are shown for epidemiological period and every week from three weeks before (−3, −2, and −1) until six weeks after (1, 2, 3, 4, 5, and 6) every sampling. Significant correlation coefficients are shown in bold.

Week	City								
	Riohacha			Bello			Villavicencio		
	DENV-1	DENV-3	DENV-4	DENV-2	DENV-3	DENV-4	DENV-1	DENV-3	DENV-4
EP †	0.096	**0.978** **	0.306	0.520	−0.460	0.062	−0.592	0.858	**0.956** *
−3	−0.461	0.668	0.073	0.750	−0.494	−0.624	−0.734	**0.992** ***	0.634
−2	−0.161	0.847	0.586	0.147	0.238	−0.585	−0.697	**0.997** ***	0.709
−1	0.183	**0.989** **	0.314	**0.907** *	−0.391	0.118	−0.669	**0.901** *	**0.914** *
0	0.210	**0.989** **	0.138	−0.147	0.556	−0.679	−0.576	**0.891** *	**0.943** *
1	0.238	**0.952** *	0.152	−0.417	−0.050	0.094	−0.589	**0.885***	**0.945** *
2	0.302	0.824	0.379	0.075	−0.385	0.311	−0.502	0.767	**0.989** **
3	0.052	0.858	0.533	0.375	0.023	0.377	−0.435	0.742	**0.997** ***
4	0.647	0.405	0.624	−0.245	0.099	−0.455	−0.479	0.811	**0.984** **
5	0.711	0.482	0.550	0.000	0.028	−0.386	−0.448	0.716	**0.995** ***
6	0.082	−0.129	**0.888** *	−0.366	0.243	−0.174	−0.367	0.773	**0.982** **

* *p*-value < 0.05; ** *p*-value < 0.01; *** *p*-value < 0.001; † EP: Epidemiological Period.

Table S7. Correlation between IR and climatic variables. Correlation coefficient for IR and different climatic variables are shown for every week from six weeks before (−6 to −1) to the same week of the samplings. Significant correlation coefficients are shown in bold.

Week	City								
	Riohacha			Bello			Villavicencio		
	RH †	Prec ‡	T° ±	RH	Prec	T°	RH	Prec	T°
−6	**0.919** *	0.680	**−0.942** *	0.114	0.327	−0.156	0.294	0.207	−0.261
−5	**0.986** **	0.626	**−0.910** *	0.486	−0.074	**−0.880** *	−0.868 ¥	−0.682	0.749
−4	**0.971** **	0.697	**−0.925** *	0.725	**0.977** **	−0.778	−0.680	−0.680	**0.889** *
−3	0.777	0.673	−0.864 ¥	0.232	0.321	−0.341	**−0.878** *	−0.032	0.830
−2	0.085	**−0.883** *	**−0.898** *	0.252	−0.136	−0.035	0.221	0.769	−0.289
−1	0.103	−0.051	−0.489	−0.030	0.652	0.073	−0.120	0.217	0.061
0	−0.128	−0.303	−0.395	0.142	−0.028	−0.231	0.213	0.875	0.112

* p-value < 0.05; ** p-value < 0.01; ¥ p-value equals to 0.05; † RH: Relative humidity (%); ‡ Prec: Precipitation (mm); ± T°: Temperature (°C).

References

1. Chutinimitkul, S.; Payungporn, S.; Theamboonlers, A.; Poovorawan, Y. Dengue typing assay based on real-time PCR using sybr green I. *J. Virol. Methods* **2005**, *129*, 8–15.
2. Shu, P.Y.; Chang, S.F.; Kuo, Y.C.; Yueh, Y.Y.; Chien, L.J.; Sue, C.L.; Lin, T.H.; Huang, J.H. Development of group- and serotype-specific one-step SYBR green I-based real-time reverse transcription-PCR assay for dengue virus. *J. Clin. Microbiol.* **2003**, *41*, 2408–2416.
3. Chien, L.J.; Liao, T.L.; Shu, P.Y.; Huang, J.H.; Gubler, D.J.; Chang, G.J. Development of real-time reverse transcriptase PCR assays to detect and serotype dengue viruses. *J. Clin. Microbiol.* **2006**, *44*, 1295–1304.
4. Seah, C.L.; Chow, V.T.; Tan, H.C.; Can, Y.C. Rapid, single-step RT-PCR typing of dengue viruses using five NS3 gene primers. *J. Virol. Methods* **1995**, *51*, 193–200.
5. Seah, C.L.; Chow, V.T.; Chan, Y.C. Semi-nested PCR using NS3 primers for the detection and typing of dengue viruses in clinical serum specimens. *Clin. Diagn. Virol.* **1995**, *4*, 113–120.

© 2016 by the authors; licensee MDPI, Basel, Switzerland. This article is an open access article distributed under the terms and conditions of the Creative Commons by Attribution (CC-BY) license (http://creativecommons.org/licenses/by/4.0/).
